# Redetermination of metarossite, CaV^5+^_2_O_6_·2H_2_O

**DOI:** 10.1107/S2056989016012433

**Published:** 2016-08-09

**Authors:** Anaïs Kobsch, Robert T. Downs, Kenneth J. Domanik

**Affiliations:** a Département des Sciences de la Terre, École Normale Supérieure de Lyon, Site Monod, 15 parvis René Descartes, BP 7000, 69342 Lyon, France; b Département des Sciences de la Terre, Université Claude Bernard Lyon 1, 43 Bd du 11 Novembre 1918, 69622 Villeurbanne Cedex, France; c Department of Geosciences, University of Arizona, 1040 E. 4th Street, Tucson, AZ 85721-0077, USA; d Lunar and Planetary Laboratory, University of Arizona, 1629 E. University Blv, Tucson, AZ 85721-0092, USA

**Keywords:** crystal structure, redetermination, metarossite, hydrogen bonds, phase transformation, brannerite

## Abstract

The crystal structure of metarossite, ideally CaV_2_O_6_·2H_2_O [chemical name: calcium divanadium(V) hexa­oxide dihydrate], was first determined using precession photographs, with fixed isotropic displacement parameters and without locating the positions of the H atoms, leading to a reliability factor *R* = 0.11 [Kelsey & Barnes (1960 ▸). *Can. Mineral.* **6**, 448–466]. This communication reports a structure redetermination of this mineral on the basis of single-crystal X-ray diffraction data of a natural sample from the Blue Cap mine, San Juan County, Utah, USA (*R*1 = 0.036). Our study not only confirms the structural topology reported in the previous study, but also makes possible the refinement of all non-H atoms with anisotropic displacement parameters and all H atoms located. The metarossite structure is characterized by chains of edge-sharing [CaO_8_] polyhedra parallel to [100] that are themselves connected by chains of alternating [VO_5_] trigonal bipyramids parallel to [010]. The two H_2_O mol­ecules are bonded to Ca. Analysis of the displacement parameters show that the [VO_5_] chains librate around [010]. In addition, we measured the Raman spectrum of metarossite and compared it with IR and Raman data previously reported. Moreover, heating of metarossite led to a loss of water, which results in a transformation to the brannerite-type structure, CaV_2_O_6_, implying a possible dehydration pathway for the compounds *M* ^2+^V_2_O_6_·*x*H_2_O, with *M* = Cu, Cd, Mg or Mn, and *x* = 2 or 4.

## Abstract 2

The redetermination of metarossite, CaV^5+^_2_O_6_·2H_2_O, based on modern single-crystal diffraction data confirms the previous study based on precession photographs, however, with the H atoms located and all atoms refined with anisotropic displacement parameters.

## Mineralogical and crystal-chemical context   

Metarossite was originally described from Bull Pen Canyon, San Miguel County, Colorado, by Foshag & Hess (1927 ▸) as a yellow, platy, soft and friable mineral with composition CaV_2_O_6_·2H_2_O. It is soluble in hot water and generally is formed as a dehydration product of rossite, CaV_2_O_6_·4H_2_O, which itself crystallizes from aqueous solutions (Ahmed & Barnes, 1963 ▸).

Barnes & Qurashi (1952 ▸) reported triclinic symmetry (*P* 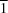) and unit-cell parameters [*a* = 6.215 (5), *b* = 7.065 (5), *c* = 7.769 (5) Å, *α* = 92.97 (17), *β* = 96.65 (17), *γ* = 105.78 (17)°] of metarossite from a sample from an area near Thompson’s, Utah. Later, by means of precession photographs, Kelsey & Barnes (1960 ▸) determined its crystal structure from the material used by Barnes & Qurashi (1952 ▸). For structure refinement (*R* = 0.11), fixed isotropic displacement parameters were introduced without locating the positions of the hydrogen atoms.

**Figure 1:**
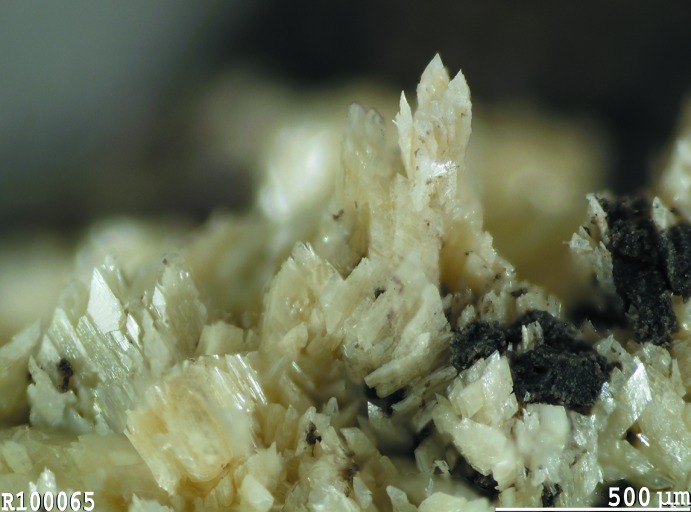
Photograph of the metarossite specimen analyzed in this study.

This study reports the refinement of the structure of a metarossite sample (Fig. 1 ▸) from the Blue Cap mine, San Juan County, Utah, USA, with anisotropic displacement parameters for all non-hydrogen atoms, positions of hydrogen atoms determined, and improvement of the reliability factor to 0.036. Raman spectra were also recorded and compared with that reported in the two studies by Frost *et al.* (2004 ▸, 2005 ▸), on a sample from the Burro mine, San Miguel County of Colorado, USA.

## Structural commentary   

**Figure 2:**
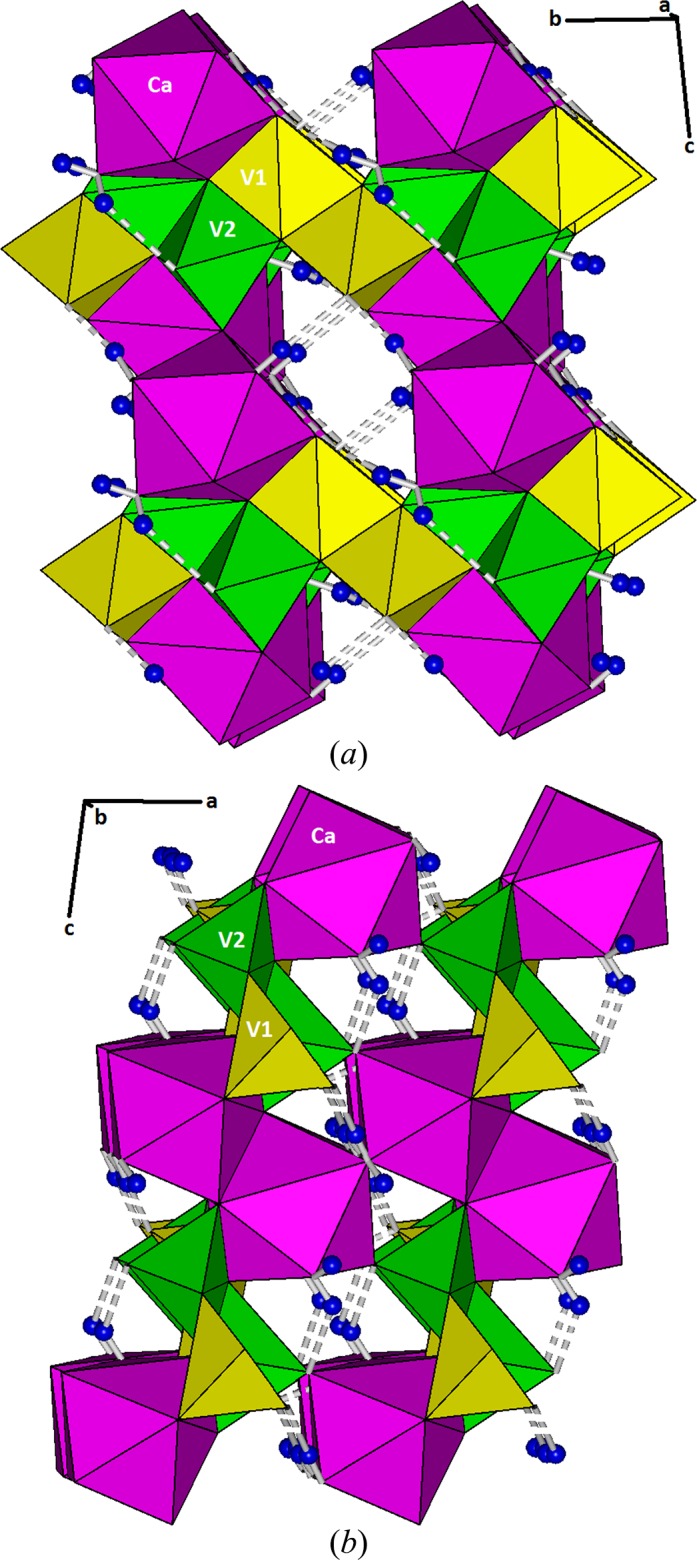
Crystal structure of metarossite, showing (*a*) the chains of alternating [V1O_5_] and [V2O_5_] trigonal bipyramids (yellow and green, respectively) along [010], and (*b*) the chains of edge-sharing distorted [CaO_8_] square anti­prisms (magenta) along [100]. H atoms are represented by blue spheres; hydrogen bonding is indicated by dashed lines.

The structural topology of metarossite for all non-hydrogen atoms from this study is identical to that reported by Kelsey & Barnes (1960 ▸). Chains of edge-sharing distorted [VO_5_] trigonal bipyramids run parallel to [010], with [V1O_5_] and [V2O_5_] polyhedra alternating along the chains (Fig. 2 ▸ *a*). These chains are linked by chains of edge-sharing [CaO_8_] polyhedra aligned parallel to [100] (Fig. 2 ▸ *b*). The water mol­ecules are located at three vertices of the [CaO_8_] polyhedra [O*W*3, O*W*8^i^ and O*W*8^ii^; symmetry codes: (i) −*x* + 1, −*y* + 1, −*z*; (ii) *x* + 1, y, *z*].

**Figure 3:**
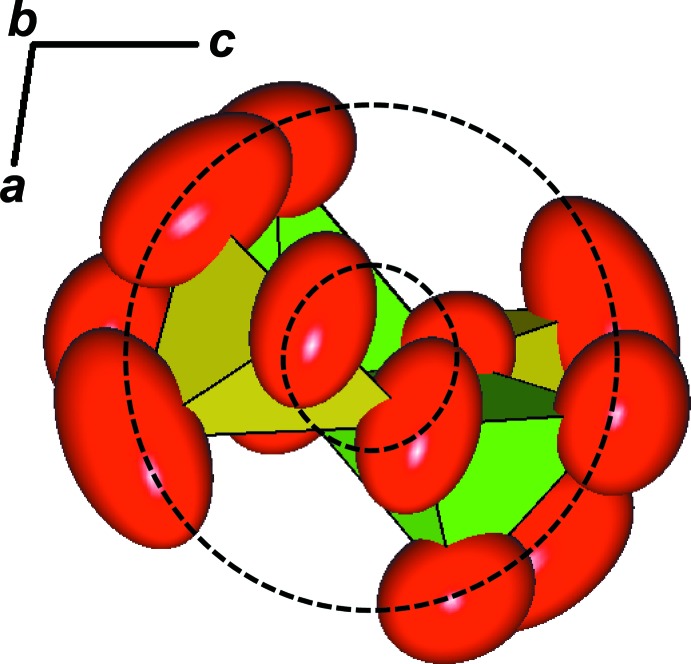
A view down [010] of the [VO_5_] chain composed of alternated [V1O_5_] (yellow) and [V2O_5_] (green) polyhedra. The red ellipsoids represent the displacement parameters of the O atoms at the 99.999% probability level. The black circles demonstrate that the entire [VO_5_] chain oscillate or librate around [010], with a slight radial increase of amplitudes.

It is inter­esting to note that there is a radial orientation of the displacement ellipsoids associated with the [VO_5_] chains when viewed along the chain direction (Fig. 3 ▸). The amplitude also slightly radially increases, as indicated by the black dashed circles in Fig. 3 ▸. We inter­pret this as the oscillation or libration of the [VO_5_] chains around [010]. A similar behavior was reported for brackebuschite Pb_2_Mn^3+^(VO_4_)_2_(OH) (Lafuente & Downs, 2016 ▸) where the [Mn^3+^(VO_4_)_2_OH] chains oscillating about an axis.

Numerical data of the hydrogen-bonding scheme in metarossite are presented in Table 1 ▸. The bond-valence calculations (Brown, 2002 ▸) with the parameters given by Brese & O’Keeffe (1991 ▸) confirm that O*W*3 and O*W*8 correspond to the two H_2_O mol­ecules (Table 2 ▸). The low bond-valence sum for O5 is because it is an acceptor for three hydrogen atoms (H2, H3 and H4; Table 1 ▸). In fact, all acceptor O atoms involved in hydrogen bonding are from VO_5_ polyhedra, providing the additional linkage between the [CaO_8_] and [VO_5_] chains.

## Raman spectrum   

**Figure 4:**
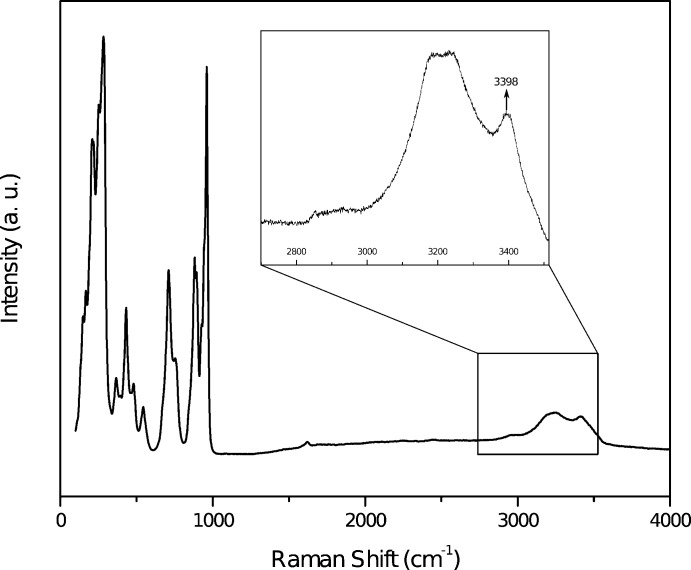
Raman spectrum of metarossite collected with a 532 nm laser. Only the band at 3400 cm^−1^ can be clearly assigned to hydrogen stretching vibrations (O*W*8–H4) but the broad shoulder discernible around 3415–3480 cm^−1^ corresponds probably to O*W*8–H3 and O*W*3–H2 vibrations.

The Raman spectrum of metarossite (Fig. 4 ▸) is comparable with the data recorded by Frost *et al.* (2005 ▸) below 1000 cm^−1^, but is different in the O–H stretching region between 2800 and 3700 cm^−1^ (Frost *et al.*, 2004 ▸). Indeed, they recorded only three Raman bands (at 3177, 3401 and 3473 cm^−1^), whereas with the present data, it is possible to distinguish four to five bands depending on the orientation (2904, 2954, 3189, 3240 and 3398 cm^−1^), along with a broad shoulder around 3415–3480 cm^−1^ (Fig. 4 ▸). According to Libowitzky (1999 ▸), the band at 3398 cm^−1^ can be attributed to the O*W*8–H4 vibration, and the broad shoulder around 3415–3480 cm^−1^ may correspond to the O*W*8–H3 and O*W*3–H2 vibrations (Table 3 ▸). The last vibration (O*W*3–H1) cannot be seen on Fig. 4 ▸, but since the frequency currently accepted for free OH^−^ ion is 3560 cm^−1^ (Lutz, 1995 ▸), it can be associated with the IR band at 3526 cm^−1^ observed by Frost *et al.* (2004 ▸).

## Synthesis and crystallization   

The natural sample used in this study is from the Blue Cap mine, San Juan County of Utah, USA (Fig. 1 ▸) and belongs to the RRUFF project collection (http://rruff.info/R100065). Chemical analysis was performed with a CAMECA SX100 electron microprobe operated at 20 kV and 20 nA and a beam size <1 µm. Eight analysis points yielded an average composition (wt.%): CaO 19.2 (1), V_2_O_5_ 66.6 (4), trace amount of Sr, and H_2_O 13.06 estimated to provide two H_2_O mol­ecules per formula unit. The empirical chemical formula, based on eight oxygen atoms, is Ca_0.94_V^5+^_2.02_O_6_·2H_2_O. The Raman spectrum of metarossite was collected from a randomly oriented crystal at 50% power of 150 mW on a Thermo–Almega microRaman system, using a solid-state laser with a wavelength of 532 nm and a thermoelectrically cooled CCD detector. The laser was partially polarized with 4 cm^−1^ resolution and a spot size of 1 µm.

## Transformation of metarossite   

**Figure 5:**
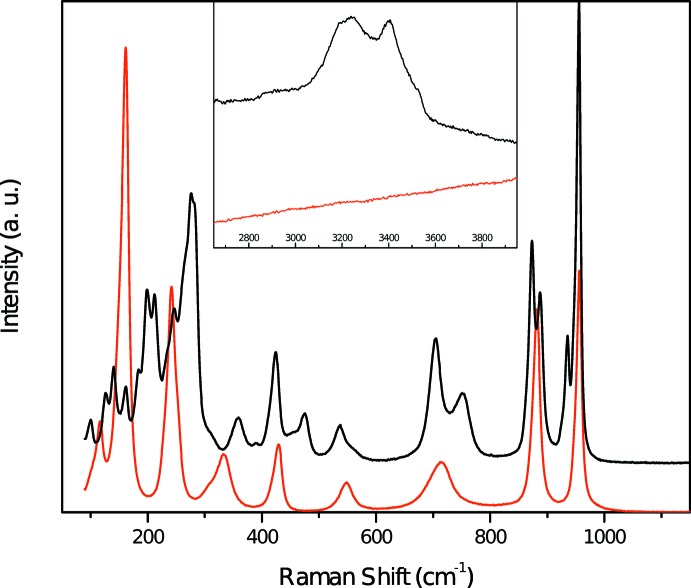
Raman spectrum of metarossite after heated by a full power 532 nm laser (red curve) and comparison with an initial metarossite spectrum (black curve).

When a small piece of metarossite (edge length in all dimensions 0.1 mm) was placed under a full power laser (150 mW, 532 nm), a change in its Raman spectrum was observed (Fig. 5 ▸). In particular, all bands originating from O–H stretching vibrations disappeared, suggesting a complete dehydration of the sample. Moreover, the spectrum below 1200 cm^−1^ was found to match that of synthetic CaV_2_O_6_ (Baran *et al.*, 1987 ▸). In addition, we observed similar Raman spectra collected from a metarossite fragment that was heated in air in an oven at 373 K for 12 h. Single crystal X-ray diffraction analysis on the heated crystal revealed monoclinic symmetry with unit cell parameters *a* = 10.0 (1), *b* = 3.6 (2), *c* = 6.9 (6) Å, *β* = 105 (6)°, which match those reported for brannerite (Szymanski & Scott, 1982 ▸). However, we were unable to obtain more detailed structure information for the heated sample due to its poor crystallinity (caused probably by dehydration).

A number of synthetic metavanadates, such as those with formula *M* ^2+^V_2_O_6_ where *M* = Cu, Cd, Mg or Mn, are found to be isostructural with brannerite (Baran *et al.*, 1987 ▸; Müller-Buschbaum & Kobel, 1991 ▸). There are also many hydrated forms of these compounds, including synthetic CuV_2_O_6_·2H_2_O (Leblanc & Ferey, 1990 ▸), and CdV_2_O_6_·2H_2_O (Ulická, 1988 ▸), as well as natural dickthomssenite MgV_2_O_6_·7H_2_O (Hughes *et al.*, 2001 ▸) or ansermetite MnV_2_O_6_·4H_2_O (Brugger *et al.*, 2003 ▸). Because tetra­hydrated or dihydrated forms of these materials have structures related to rossite or metarossite, it is likely, then, that natural equivalents of the synthetic metavanadates *M* ^2+^V_2_O_6_·*x*H_2_O (*M* = Cu, Cd, Mg or Mn and *x* = 0, 2 or 4) may exist.

## Refinement details   

Crystal data, data collection and structure refinement details are summarized in Table 4 ▸. The electron microprobe analysis revealed traces of Sr in our sample. The empirical formula shows a little deficiency for Ca and excess for V. For simplicity, the ideal chemical formula CaV_2_O_6_·2H_2_O was assumed during the refinement. Kelsey & Barnes (1960 ▸) underline that {101} is often a twin-plane in metarossite, but the crystal used for this X-ray analysis did not show twinning. Atomic coordinates of the previous study were taken as starting parameters for refinement. The H atoms were located from difference Fourier syntheses and their positions refined with fixed isotropic displacement parameters (*U*_iso_ = 0.04 Å^2^). The maximum residual electron density in the difference Fourier maps was located at 0.86 Å from O7 and the minimum density at 1.39 Å from Ca.

## Supplementary Material

Crystal structure: contains datablock(s) I. DOI: 10.1107/S2056989016012433/wm5311sup1.cif

Structure factors: contains datablock(s) I. DOI: 10.1107/S2056989016012433/wm5311Isup2.hkl

CCDC reference: 1497229

Additional supporting information: crystallographic information; 3D view; checkCIF report

## Figures and Tables

**Table 1:** Hydrogen-bond geometry (Å, °)

*D*—H⋯*A*	*D*—H	H⋯*A*	*D*⋯*A*	*D*—H⋯*A*
O*W*3—H1⋯O4^i^	0.78 (5)	2.37 (5)	2.965 (4)	133 (4)
O*W*3—H2⋯O5^ii^	0.72 (4)	2.25 (5)	2.900 (3)	150 (5)
O*W*8—H3⋯O5^iii^	0.75 (4)	2.09 (5)	2.810 (3)	162 (5)
O*W*8—H4⋯O5^iv^	0.84 (4)	1.97 (5)	2.794 (3)	166 (4)

Symmetry codes: (i) 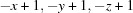; (ii) 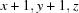; (iii) 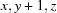; (iv) 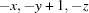.

**Table 2:** Bond-valence sums for metarossite

	O1	O2	O*W*3*	O4	O5	O6	O7	O*W*8*	**Σ_M_**
Ca		0.253	0.218	0.315		0.272	0.240	0.205	**2.107**
							0.326	0.278	
V1	0.581	0.687			1.491	1.624			**5.153**
	0.770								
V2	0.825	0.780		1.584			1.415		**5.083**
		0.479							
**Σ_O_**	**2.176**	**2.200**	**0.218**	**1.899**	**1.491**	**1.897**	**1.981**	**0.482**	

Note: (*) H atoms not considered for calculation.

**Table 3:** O⋯O measured distances (Å), Raman stretching frequencies (cm^−1^) calculated using the correlation for *d* < 3.2 Å and samples without Cu (Libowitzky, 1999 ▸), and comparison with O⋯O calculated by Frost *et al.* (2004 ▸) from IR frequencies (cm^−1^).

	This study		Frost *et al.* (2004 ▸)	
O—H⋯O	O⋯O	ν	ν	O⋯O
O*W*3—H1⋯O4	2.965	3504	3526	2.9393
O*W*3—H2⋯O5	2.900	3482	3387	2.7995
O*W*8—H3⋯O5	2.810	3421	3181	2.6977
O*W*8—H4⋯O5	2.794	3404	2867	2.6227

**Table 4:** Experimental details

Crystal data	
Chemical formula	CaV_2_O_6_·2H_2_O
*M*_r_	273.99
Crystal system, space group	Triclinic, *P* 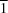
Temperature (K)	293
*a*, *b*, *c* (Å)	6.2059 (4), 7.0635 (4), 7.7516 (5)
α, β, γ (°)	93.166 (4), 96.548 (4), 105.883 (4)
*V* (Å^3^)	323.36 (4)
*Z*	2
Radiation type	Mo *K*α
μ (mm^−1^)	3.68
Crystal size (mm)	0.07 × 0.07 × 0.06

Data collection	
Diffractometer	Bruker APEXII CCD area-detector
Absorption correction	Multi-scan (*SADABS*; Bruker, 2004 ▸)
*T*_min_, *T*_max_	0.669, 0.746
No. of measured, independent and observed [*I* > 2σ(*I*)] reflections	5576, 2075, 1508
*R*_int_	0.037
(sin θ/λ)_max_ (Å^−1^)	0.735

Refinement	
*R*[*F* ^2^ > 2σ(*F* ^2^)], *wR*(*F* ^2^), *S*	0.036, 0.076, 1.01
No. of reflections	2075
No. of parameters	113
H-atom treatment	Only H-atom coordinates refined
Δρ_max_, Δρ_min_ (e Å^−3^)	0.77, −0.58

Computer programs: *APEX2* and *SAINT* (Bruker, 2004 ▸), *SHELXT* (Sheldrick, 2015*a* ▸), *SHELXL2014* (Sheldrick, 2015*b* ▸), *XtalDraw* (Downs & Hall-Wallace, 2003 ▸) and *publCIF* (Westrip, 2010 ▸).

## supplementary crystallographic information

### Crystal data

**Table:**

CaV_2_O_6_·2H_2_O	*Z* = 2
*M**_r_* = 273.99	*F*(000) = 268
Triclinic, *P*1	*D*_x_ = 2.814 Mg m^−^^3^
*a* = 6.2059 (4) Å	Mo *K*α radiation, λ = 0.71073 Å
*b* = 7.0635 (4) Å	Cell parameters from 1255 reflections
*c* = 7.7516 (5) Å	θ = 2.7–29.8°
α = 93.166 (4)°	µ = 3.68 mm^−^^1^
β = 96.548 (4)°	*T* = 293 K
γ = 105.883 (4)°	Platy, pale yellow
*V* = 323.36 (4) Å^3^	0.07 × 0.07 × 0.06 mm

### Data collection

**Table:**

Bruker APEXII CCD area-detector diffractometer	1508 reflections with *I* > 2σ(*I*)
φ and ω scan	*R*_int_ = 0.037
Absorption correction: multi-scan (*SADABS*; Bruker, 2004)	θ_max_ = 31.5°, θ_min_ = 2.7°
*T*_min_ = 0.669, *T*_max_ = 0.746	*h* = −9→8
5576 measured reflections	*k* = −10→10
2075 independent reflections	*l* = −11→11

### Refinement

**Table:**

Refinement on *F*^2^	Hydrogen site location: difference Fourier map
Least-squares matrix: full	Only H-atom coordinates refined
*R*[*F*^2^ > 2σ(*F*^2^)] = 0.036	*w* = 1/[σ^2^(*F*_o_^2^) + (0.0318*P*)^2^ + 0.0696*P*] where *P* = (*F*_o_^2^ + 2*F*_c_^2^)/3
*wR*(*F*^2^) = 0.076	(Δ/σ)_max_ < 0.001
*S* = 1.01	Δρ_max_ = 0.77 e Å^−^^3^
2075 reflections	Δρ_min_ = −0.58 e Å^−^^3^
113 parameters	Extinction correction: SHELXL2014 (Sheldrick, 2015b), Fc^*^=kFc[1+0.001xFc^2^λ^3^/sin(2θ)]^-1/4^
0 restraints	Extinction coefficient: 0.005 (2)

### Special details

**Table:**

Geometry. All esds (except the esd in the dihedral angle between two l.s. planes) are estimated using the full covariance matrix. The cell esds are taken into account individually in the estimation of esds in distances, angles and torsion angles; correlations between esds in cell parameters are only used when they are defined by crystal symmetry. An approximate (isotropic) treatment of cell esds is used for estimating esds involving l.s. planes.

### Fractional atomic coordinates and isotropic or equivalent isotropic displacement parameters (Å^2^)

**Table:**

	*x*	*y*	*z*	*U*_iso_*/*U*_eq_	
Ca	0.76199 (10)	0.46286 (9)	0.14933 (8)	0.01285 (14)	
V1	0.44824 (9)	0.10219 (7)	0.33438 (6)	0.01068 (13)	
V2	0.37329 (8)	0.58264 (7)	0.34629 (6)	0.01004 (13)	
O1	0.4051 (4)	0.8480 (3)	0.4170 (3)	0.0137 (4)	
O2	0.5250 (3)	0.3869 (3)	0.3867 (3)	0.0106 (4)	
OW3	0.8519 (5)	0.7632 (4)	0.3671 (4)	0.0236 (6)	
O4	0.1030 (3)	0.4726 (3)	0.3330 (3)	0.0157 (5)	
O5	0.1896 (4)	0.0684 (3)	0.2322 (3)	0.0207 (5)	
O6	0.6214 (4)	0.1115 (3)	0.1910 (3)	0.0232 (5)	
O7	0.4308 (4)	0.5998 (3)	0.1408 (3)	0.0155 (5)	
OW8	0.0045 (4)	0.7225 (3)	0.0059 (3)	0.0164 (5)	
H1	0.937 (8)	0.747 (6)	0.444 (6)	0.040*	
H2	0.900 (8)	0.856 (7)	0.332 (6)	0.040*	
H3	0.078 (8)	0.808 (7)	0.066 (6)	0.040*	
H4	−0.069 (7)	0.766 (6)	−0.073 (6)	0.040*	

### Atomic displacement parameters (Å^2^)

**Table:**

	*U*^11^	*U*^22^	*U*^33^	*U*^12^	*U*^13^	*U*^23^
Ca	0.0120 (3)	0.0142 (3)	0.0116 (3)	0.0024 (2)	0.0017 (2)	0.0017 (2)
V1	0.0140 (3)	0.0077 (2)	0.0094 (2)	0.00209 (19)	0.00032 (18)	0.00023 (18)
V2	0.0124 (2)	0.0082 (2)	0.0087 (2)	0.0026 (2)	−0.00078 (18)	−0.00002 (18)
O1	0.0216 (11)	0.0084 (10)	0.0098 (10)	0.0030 (9)	−0.0002 (8)	0.0005 (8)
O2	0.0116 (10)	0.0092 (10)	0.0109 (10)	0.0032 (8)	−0.0001 (8)	0.0002 (8)
OW3	0.0237 (13)	0.0201 (13)	0.0243 (14)	0.0026 (11)	0.0011 (10)	0.0030 (11)
O4	0.0131 (10)	0.0160 (11)	0.0162 (11)	0.0021 (9)	−0.0006 (8)	0.0013 (9)
O5	0.0211 (12)	0.0136 (11)	0.0232 (13)	0.0024 (9)	−0.0075 (9)	0.0003 (9)
O6	0.0347 (14)	0.0147 (11)	0.0204 (12)	0.0027 (11)	0.0151 (10)	0.0004 (9)
O7	0.0188 (11)	0.0170 (11)	0.0113 (10)	0.0064 (9)	0.0007 (8)	0.0020 (9)
OW8	0.0196 (13)	0.0132 (12)	0.0130 (11)	0.0005 (10)	−0.0014 (9)	0.0006 (9)

### Geometric parameters (Å, º)

**Table:**

Ca—O7^i^	2.381 (2)	V1—O1^iii^	1.899 (2)
Ca—O4^ii^	2.394 (2)	V1—O2	1.943 (2)
Ca—OW8^ii^	2.441 (2)	V1—O1^iv^	2.004 (2)
Ca—O6	2.448 (2)	V1—V1^v^	3.1033 (10)
Ca—O2	2.476 (2)	V1—V2^iv^	3.1187 (7)
Ca—O7	2.496 (2)	V2—O4	1.633 (2)
Ca—OW3	2.530 (3)	V2—O7	1.675 (2)
Ca—OW8^i^	2.554 (2)	V2—O1	1.874 (2)
V1—O6	1.623 (2)	V2—O2	1.895 (2)
V1—O5	1.655 (2)	V2—O2^iv^	2.075 (2)
			
O7^i^—Ca—O4^ii^	145.60 (8)	O6—Ca—OW8^i^	71.80 (7)
O7^i^—Ca—OW8^ii^	79.03 (8)	O2—Ca—OW8^i^	133.65 (7)
O4^ii^—Ca—OW8^ii^	84.88 (8)	O7—Ca—OW8^i^	149.58 (7)
O7^i^—Ca—O6	89.84 (8)	OW3—Ca—OW8^i^	135.12 (8)
O4^ii^—Ca—O6	88.49 (8)	O6—V1—O5	109.11 (13)
OW8^ii^—Ca—O6	148.85 (8)	O6—V1—O1^iii^	105.21 (11)
O7^i^—Ca—O2	116.63 (7)	O5—V1—O1^iii^	97.89 (11)
O4^ii^—Ca—O2	93.49 (7)	O6—V1—O2	94.99 (10)
OW8^ii^—Ca—O2	145.90 (8)	O5—V1—O2	97.89 (10)
O6—Ca—O2	64.79 (7)	O1^iii^—V1—O2	148.57 (9)
O7^i^—Ca—O7	72.07 (8)	O6—V1—O1^iv^	114.83 (11)
O4^ii^—Ca—O7	140.72 (7)	O5—V1—O1^iv^	135.88 (11)
OW8^ii^—Ca—O7	97.51 (8)	O1^iii^—V1—O1^iv^	74.72 (9)
O6—Ca—O7	106.65 (8)	O2—V1—O1^iv^	75.01 (8)
O2—Ca—O7	63.17 (7)	O4—V2—O7	106.24 (11)
O7^i^—Ca—OW3	131.12 (9)	O4—V2—O1	105.17 (10)
O4^ii^—Ca—OW3	72.51 (9)	O7—V2—O1	100.84 (10)
OW8^ii^—Ca—OW3	76.53 (8)	O4—V2—O2	106.81 (10)
O6—Ca—OW3	129.97 (8)	O7—V2—O2	93.48 (10)
O2—Ca—OW3	70.58 (8)	O1—V2—O2	139.47 (9)
O7—Ca—OW3	70.05 (8)	O4—V2—O2^iv^	102.14 (10)
O7^i^—Ca—OW8^i^	77.53 (7)	O7—V2—O2^iv^	151.38 (10)
O4^ii^—Ca—OW8^i^	69.34 (7)	O1—V2—O2^iv^	74.79 (8)
OW8^ii^—Ca—OW8^i^	77.39 (8)	O2—V2—O2^iv^	74.69 (9)

Symmetry codes: (i) −*x*+1, −*y*+1, −*z*; (ii) *x*+1, *y*, *z*; (iii) *x*, *y*−1, *z*; (iv) −*x*+1, −*y*+1, −*z*+1; (v) −*x*+1, −*y*, −*z*+1.

### Hydrogen-bond geometry (Å, º)

**Table:**

*D*—H···*A*	*D*—H	H···*A*	*D*···*A*	*D*—H···*A*
O*W*3—H1···O4^iv^	0.78 (5)	2.37 (5)	2.965 (4)	133 (4)
O*W*3—H2···O5^vi^	0.72 (4)	2.25 (5)	2.900 (3)	150 (5)
O*W*8—H3···O5^vii^	0.75 (4)	2.09 (5)	2.810 (3)	162 (5)
O*W*8—H4···O5^viii^	0.84 (4)	1.97 (5)	2.794 (3)	166 (4)

Symmetry codes: (iv) −*x*+1, −*y*+1, −*z*+1; (vi) *x*+1, *y*+1, *z*; (vii) *x*, *y*+1, *z*; (viii) −*x*, −*y*+1, −*z*.
